# CD312 Promotes Paediatric Acute Lymphoblastic Leukaemia Through GNA15‐Mediated Non‐Classical GPCR Signalling Pathway

**DOI:** 10.1111/jcmm.70283

**Published:** 2024-12-10

**Authors:** Yaping Wang, Jiali Wang, Xiaopeng Ma, Huimin Li, Xiaoyan Sun, Meiyun Kang, Heng Zhang, Yao Xue, Yongjun Fang

**Affiliations:** ^1^ Department of Hematology and Oncology, Children's Hospital of Nanjing Medical University Nanjing Medical University Nanjing China

**Keywords:** acute lymphoblastic leukaemia, CD312, GPCR, immune microenvironment

## Abstract

The bone marrow‐infiltrated immune microenvironment plays a crucial role in blood system diseases, such as leukaemia. In this study, we aimed to investigate the critical role of the immune microenvironment in the onset and progression of childhood acute lymphoblastic leukaemia (ALL). Through high‐throughput detection and screening of the GPCR database in the childhood ALL immune microenvironment, we identified CD312 as a candidate target. CD312 is associated with the distribution of Treg and CTL cells within the bone marrow immune microenvironment of ALL children. After CD312 knockdown, the proportion of the Treg subgroup in immune cells was significantly reduced, whereas the proportion of CTL subgroup cells was increased. CD312 exhibited good affinity with GNA15 in the transmembrane intracellular segment, and it could interact with GNA15. The BrdU staining assay revealed that the proliferation of leukaemia cells was enhanced in the CD312‐overexpressed CD3+ T cells group via the phosphorylation of ERK, JNK and p38, whereas it was decreased by GNA15 knockdown in the co‐culture system. In conclusion, our study suggests that CD312 fosters a suppressive immune microenvironment in the onset and progression of paediatric ALL through a GNA15‐mediated non‐classical GPCR signalling pathway.

**Abbreviations**
ALL: acute lymphoblastic leukaemia
TME: tumour microenvironment
CAF: cancer‐associated fibroblasts
GPCR: G protein‐coupled receptor
CTL: cytotoxic T lymphocyte
PLA: proximity ligation assay
qRT‐PCR: quantitative real‐time polymerase chain reaction

## 1 Introduction

Acute lymphoblastic leukaemia (ALL) in children is a malignant clonal disease characterised by abnormal proliferation and differentiation apoptosis of lymphocytes [1, 2], accounting for 75% of all childhood leukaemia [3]. With the improvement of diagnosis and treatment, the 5‐year overall survival (OS) rate has exceeded 80%–90% by application of drug therapy and haematopoietic stem cell transplantation [4, 5]. However, a large number of ALL patients still die from chemotherapy complications and recurrence. Therefore, investigating specific targets and their molecular mechanisms to identify the new targeted therapy has become a hotspot.

In recent years, tumour microenvironment (TME) has been investigated and plays a crucial role in tumour progress and drug resistance [6, 7]. The microenvironment mainly includes the extracellular matrix, tumour‐associated fibroblasts (CAF), macrophages, lymphocytes and others [8, 9]. Especially, the immune microenvironment composed of macrophages, lymphocytes and others plays an important role in the occurrence and development of tumours. Previous studies have found that the abnormal distribution of CD4^+^CD25^+^ regulatory T cells in the peripheral blood of leukaemia patients had an inhibitory effect on the immune microenvironment [10]. In addition, CD8^+^ T cells could recognise tumour antigens presented by class I MHC molecules on the surface of tumour cells, thereby initiating the killing of target cells [11]. Cells can effectively bind to target cells. Therefore, uncovering the immune microenvironment and molecular regulatory mechanisms in childhood ALL is important to prevent and treat childhood ALL.

The G protein‐coupled receptor (GPCR) is widely distributed in the central nervous system [12], immune system [13], cardiovascular [14], retina and other organs and tissues, participating in tissue development and function. GPCR has always been the most widely used drug target in drug development, because about 50% of the commercially available drugs directly act on GPCR. CD312, also known as ADGRE2 or EMR2, is one of the adhesive GPCR‐related molecules in the GPCR family [15]. Patients with CD312 gene mutation can activate intracellular signalling pathways, leading to the activation and degranulation of mast cells [16]. However, there is no clear research report on the regulatory role of CD312 in human tumour diseases, especially in leukaemia and leukaemia related immune microenvironments. In our study, we showed that the level of CD312 expression was decreased in CD8^+^T cells, which was increased in CD4^+^ T cells. The expression of CD312 is associated with the distribution of regulatory T cells (Treg) and cytotoxic T lymphocytes (CTL) in the bone marrow immune microenvironment of children with ALL. Moreover, CD312 could bind to GNA15 in CD3 + T cells to promote the proliferation in leukaemia cells through activating the phosphorylation of ERK, JNK and p38 pathways.

## 2 Materials and Methods

### 2.1 Clinical Samples

Three pairs of bone marrow samples from children with ALL and three bone marrow samples from children without non‐tumour diseases were obtained from the Department of Haematology and Oncology, Children's Hospital of Nanjing Medical University. This study was approved by the Ethics Committee of Children's Hospital of Nanjing Medical University. Informed consent was obtained and signed by the guardians of the patients.

### 2.2 Flow Cytometry

Lymphocyte markers CD8a (HIT8a), CD4 (OKT4) and CD3e (UCHT1) antibodies were purchased from BD Biosciences. Treated cells were washed three times with FACS buffer containing 2 mM EDTA, 0.01% sodium azide and 5% fetal bovine serum (FBS). Cells were then incubated with antibodies for 30 min. After washing, the cells were analysed using an Attune NXT Flow cytometer (ThermoFisher).

### 2.3 Immunofluorescence Assay

Treated cells were fixed with 4% paraformaldehyde for 15 min, permeabilised with 0.1% Triton X‐100 (Beyotime, China) and blocked with 10% FBS (Beyotime, China) at room temperature. Tissues and cells were incubated with primary antibodies overnight at 4°C, and secondary antibodies were incubated the following day.

### 2.4 Coimmunoprecipitation

Treated cells were lysed with buffer containing 40 mM HEPES (pH 7.4), 0.5% Triton X‐100, 2 mM EDTA and protease inhibitors. The lysates were subjected to low‐speed rotation and then centrifuged at 12,000 × g for 10 min. The supernatant was incubated with the antibody overnight at 4°C and with A/G agarose beads for 4 h the next day. All samples were separated by sodium dodecyl sulfate–polyacrylamide gel electrophoresis (SDS‐PAGE) and detected with antibodies.

### 2.5 Cell Proliferation Detection

Treated cells were seeded on an insert membrane, and leukaemia cells were placed on the well surface to establish a co‐culture system for 48 h. A CCK8 assay kit (Sigma, USA) was used to measure proliferation in Huh7 cells. Absorbance was measured at 450 nm using a microplate reader.

### 2.6 TUNEL Staining

The rate of apoptosis was determined using a TUNEL assay with a detection kit (Boehringer Mannheim, Germany) according to the manufacturer's instructions. The apoptotic index was calculated as the number of apoptotic cells divided by the total number of cells.

### 2.7 Quantification of Apoptosis via Flow Cytometry

Treated cells were collected and fixed in 70% ethanol at 4°C. Cells were incubated with Annexin V‐FITC and 7‐AAD at 37°C for 15 min. After washing, the cells were analysed using an Attune NXT Flow cytometer (ThermoFisher).

### 2.8 Proximity Ligation Assay (PLA) Assay

An in situ PLA was performed using a DuoLink PLA kit from Sigma‐Aldrich according to the manufacturer's protocol. Briefly, treated cells were fixed with 4% paraformaldehyde and washed with PBS. Cells were blocked with Duolink block solution, and primary antibodies were incubated overnight. PLA probes were added, followed by incubation with secondary antibodies, ligation mix and amplification mix.

### 2.9 Western Blotting Analysis

Protein was extracted using RIPA lysis buffer (Beyotime, China). Protein concentration was measured using a BCA kit (Thermo, USA). Western blotting was performed, and the following antibodies were used: CD312 (#4443S), Anti‐GNA15 (ab225949), p38 (#8690), p‐p38 (#4511), JNK (#9252), p‐JNK (#9255), ERK (#4695), p‐ERK (#4370) and GAPDH (#4511) from Cell Signalling Technology (CST, USA). Bands were visualised using chemiluminescent substrate (ECL; Millipore, USA), and results were analysed using Image Lab software.

### 2.10 Statistical Analysis

Data are presented as mean ± standard error of the mean (SEM). Differences between two groups were analysed using Student's *t*‐test. One‐way analysis of variance (ANOVA) was used to determine significant differences among three or more groups. Correlations were analysed using Pearson correlation coefficients. Statistical significance was set at *p* < 0.05. Statistical analysis was performed using MedCalc 16.2.0 (MedCalc Software bvba, Ostend, Belgium), and graphs were generated using GraphPad Prism 7.0 (GraphPad Software, San Diego, CA) and R 3.5.0 (R Development Core Team, https://cran.r‐project.org/).

## 3 Results

### 3.1 Differentially Expressed GPCRs in ALL Children

**FIGURE 1:**
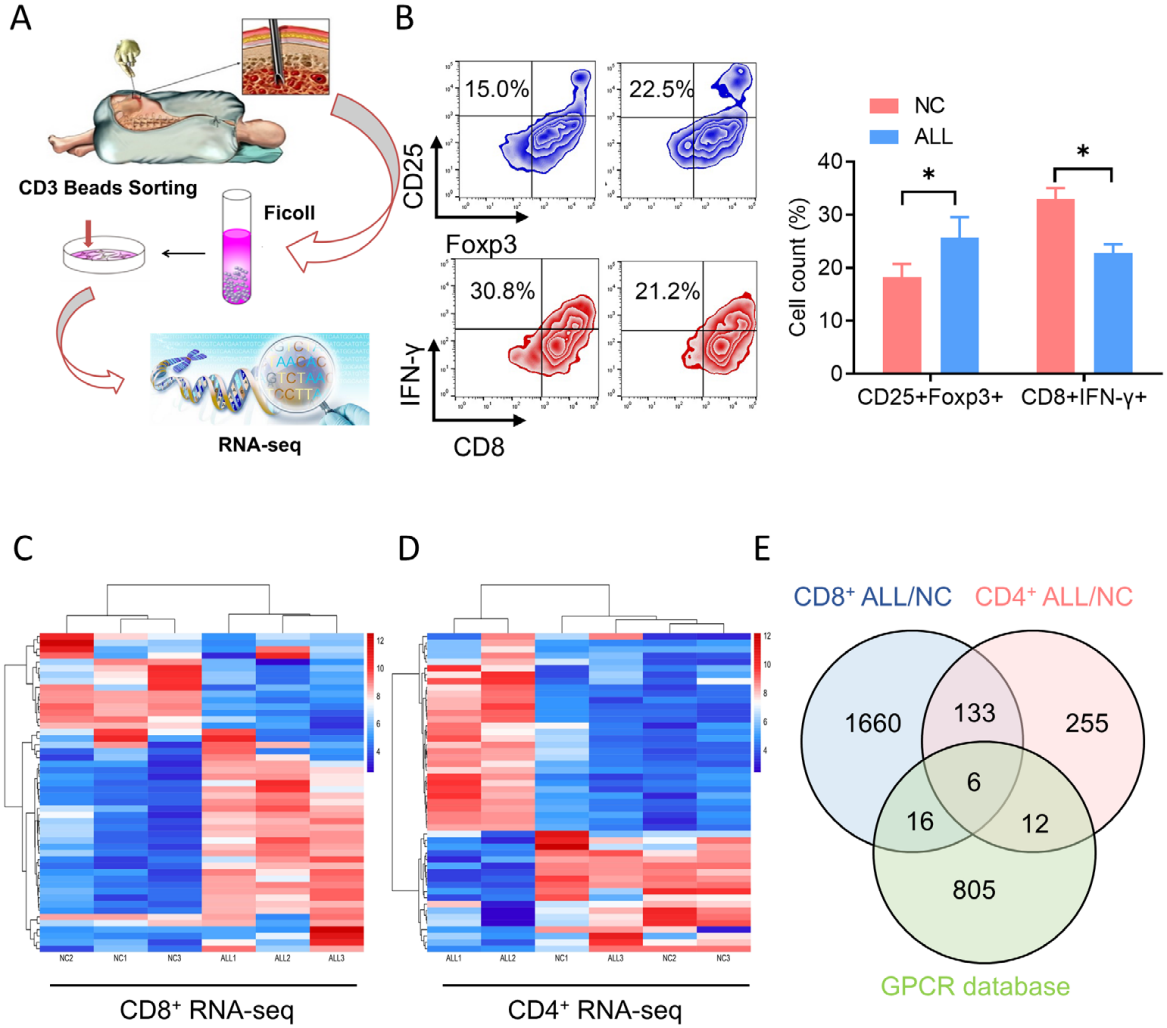
The expression of GPCRs in ALL children. (A) Experimental design. Three bone marrow samples from ALL children selected as patients group samples, three bone marrow samples from children without non‐tumour diseases were used as controls. (B) Flow cytometry was used to observe the distribution of Treg cell subpopulations and CTL cell subpopulations in the bone marrow. *n* = 5. (C, D) Heatmap of differentially genes in CD8+ T cells (C) and CD4+ T cells(C) by the high‐throughput screening. (E) 6 GPCRs exhibited differential expression in both CD8+ and CD4+ cells. *p < 0.05.

As shown in Figure 1A, three bone marrow samples from children with ALL were selected as patient group samples, and three bone marrow samples from children without non‐tumour diseases were used as controls. Infiltrating lymphocytes were isolated and expanded in vitro with IL‐2 for 72 h. CD8+ and CD4+ immune cells were then obtained using CD8/CD4‐specific magnetic beads. High‐throughput transcriptome sequencing was used to identify differentially expressed genes between the two groups. First, we used flow cytometry to observe the distribution of Treg cell subpopulations and CTL cell subpopulations in the bone marrow. The results indicated that the proportion of Treg cells in the bone marrow of children with ALL was significantly higher than in normal children, while the proportion of CTL cells was lower, indicating an immunosuppressive state in children with ALL (Figure 1B). High‐throughput screening revealed 1815 differentially expressed mRNAs in CD8+ T cells compared to healthy controls (918 upregulated and 897 downregulated); 403 differentially expressed mRNAs were found in CD4+ T cells (157 upregulated and 246 downregulated) (Figure 1C). Six GPCRs exhibited differential expression in both CD8+ and CD4+ T cells (Figure 1D).

### 3.2 CD312 Was Related to the Immunosuppressive State

**FIGURE 2:**
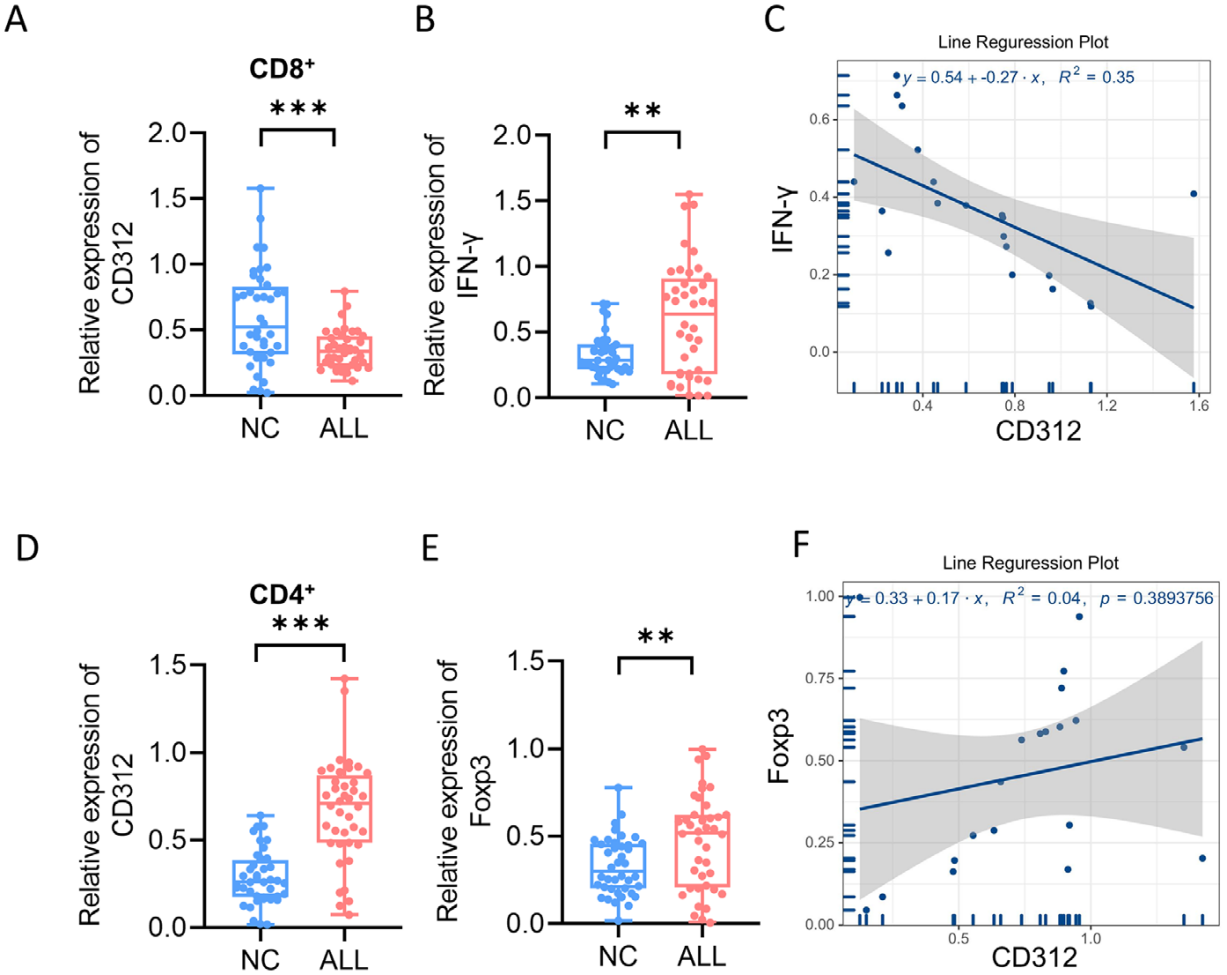
CD312 expression was related to immunosuppressive state. (A) The expression of CD312 in both CD8^+^ T cells was detected by RT‐PCR (*n* = 40). (B) The expression of IFN‐ γ in CD8^+^ T cells was detected by RT‐PCR (*n* = 40). (C) Pearson correlation analysis indicated that CD312 expression was a negative correlation with IFN‐ γ. (D) The expression of CD312 in both CD4^+^ T cells was detected by RT‐PCR (*n* = 40). (E) The expression of IFN‐ γ in CD4^+^ T cells was detected by RT‐PCR (*n* = 40). (F) Pearson correlation analysis indicated that CD312 expression was a negative correlation with Foxp3. *p < 0.05, **p < 0.01, *p < 0.001.

We detected the expression of CD312 in both CD8+ T cells and CD4+ T cells. The results showed that the level of CD312 expression was decreased in CD8+ T cells (Figure 2A). Reverse transcriptase polymerase chain reaction (RT‐PCR) was performed to detect the expression of IFN‐γ (Figure 2B). The results showed that the expression of IFN‐γ was decreased in CD3+ T cells from children with ALL. Pearson correlation analysis indicated that CD312 expression was negatively correlated with IFN‐γ (Figure 2C). Additionally, we detected the expression of CD312 in CD4+ T cells, and the results showed that CD312 expression was upregulated (Figure 2D). Expression of Foxp3 was also increased in CD3+ T cells from children with ALL (Figure 2E). Pearson correlation analysis showed that CD312 expression was positively correlated with the Foxp3 expression (Figure 2F). These results indicated that the expression of CD312 was related to the immunosuppressive state in children with ALL. We also detected the expression of five other GPCRs. There was no significant statistical difference in FZD4, GPR135 and HRH1 between children with ALL and control groups. However, LAPR1 and MTNR18 levels were higher in CD4+ T cells from children with ALL (Figure S1).

### 3.3 CD312 Was Related to the Distribution of Treg and CTL Cells

**FIGURE 3:**
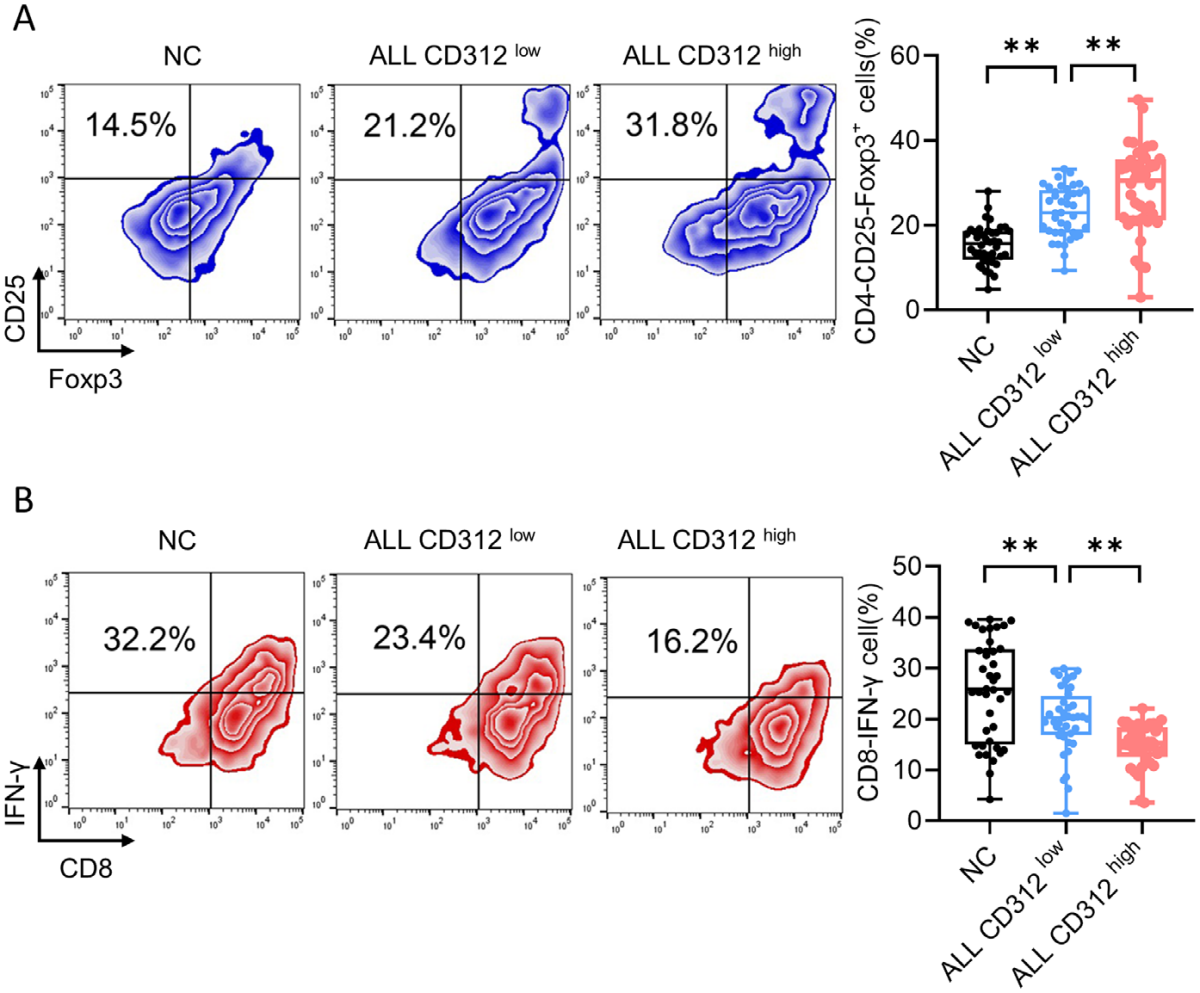
CD312 was related to the distribution of Treg and CTL cells. (A, B) The proportion of Treg cells was detected by flow cytometry in the normal control group, CD312 high expression group (CD312 high) and CD312 low expression group (CD312 low). *n* = 40. (C, D) The proportion of CTL cells was detected by flow cytometry in the normal control group, CD312 high expression group (CD312 high) and CD312 low expression group (CD312 low). *n* = 40. **p < 0.01.

Based on the expression of CD312 in bone marrow samples from children with ALL, we divided the 65 children into a CD312 high‐expression group (CD312 high) and a CD312 low‐expression group (CD312 low) according to the median expression level of CD312. Combined with the normal control group, the proportion of Treg cells was higher in the CD312 low group, while it was highest in the CD312 high group (Figure 3A,B). We then detected the proportion of CTL cells in different groups. The results indicated that the proportion of CTL cells was lower in the CD312 low group compared to the normal control group, and the proportion of CTL cells was further decreased in the CD312 high group (Figure 3C,D). These results indicated that the expression of CD312 is related to the distribution of Treg and CTL cells in the bone marrow immune microenvironment of children with ALL.

### 3.4 CD312 Knockdown Disturbed Treg and CTL Cell Subpopulations

**FIGURE 4:**
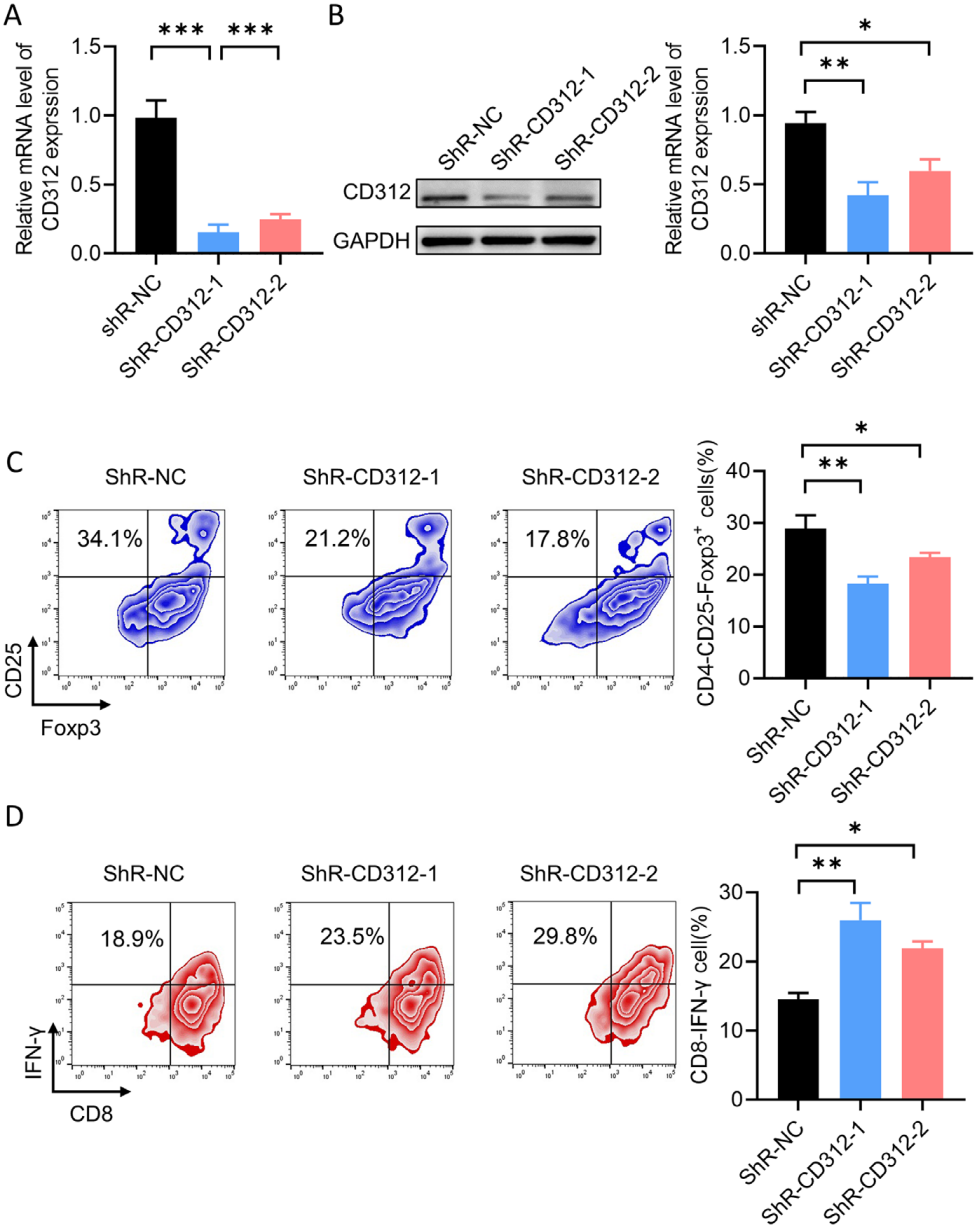
CD312 knockdown regulated Treg and CTL cells distribution. (A, B) The RT‐PCR and western blot assays were used to detect the expression of the CD312 level in the CD312 shRNA group. *n* = 3. (C, D) The distribution of Treg and CTL in the cell subpopulations was detected by flow cytometry after CD312 knockdown. *n* = 3. *p < 0.05, **p < 0.01.

We used shRNA to knock down the expression of CD312 in CD3+ T cells. First, we confirmed the knockdown efficiency of shRNA. RT‐PCR and western blot assays indicated that the expression of CD312 was significantly lower in the CD312 shRNA group (Figure 4A,B). We then detected the distribution of Treg and CTL cell subpopulations by flow cytometry. As shown in Figure 4C,D, the proportion of the Treg subgroup in immune cells was significantly downregulated, while the proportion of the CTL subgroup cells was increased after CD312 knockdown.

### 3.5 CD312 in CD3+ T Cells Promoted the Proliferation of Leukaemia Cells

**FIGURE 5:**
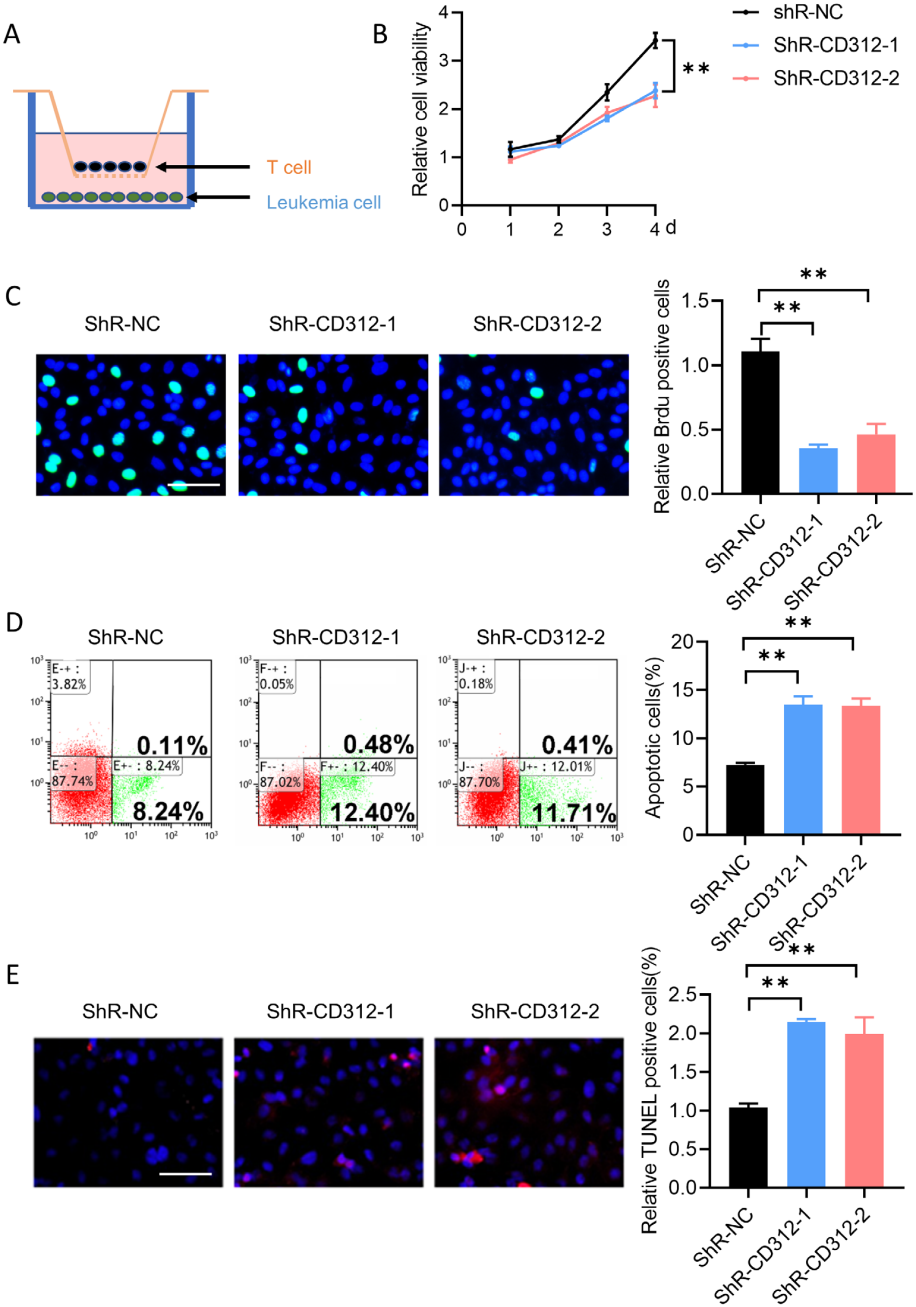
CD312 in CD3 + T cells promoted the proliferation of leukaemia cells. (A) The design of the co‐culture system. (B, C) The CCK8 assay and BrdU staining were conducted to evaluate the proliferation of leukaemia cells after CD312 knockdown in CD3 + T cells. *n* = 3. (D, E) The flow cytometry and TUNEL staining were conducted to evaluate the apoptosis of leukaemia cells after CD312 knockdown in CD3 + T cells. *n* = 3. **p < 0.01.

We further investigated the effects of CD312 in CD3+ T cells on leukaemia cells. A co‐culture system was constructed using a Transwell assay (Figure 5A). We knocked down the expression of CD312 in CD3+ T cells and cultured the treated cells in the upper chamber. The CCK8 assay was conducted to evaluate the proliferation of leukaemia cells. The results showed that the viability of leukaemia cells was decreased in the CD312 knockdown group (Figure 5B). Additionally, the proliferation of leukaemia cells was downregulated in the CD312 knockdown group (Figure 5C). Cell apoptosis of leukaemia cells was detected by flow cytometry and TUNEL staining. The results showed that cell apoptosis was increased in the CD312 knockdown group (Figure 5D,E). These results indicated that the expression of CD312 in CD3+ T cells could promote the proliferation of leukaemia cells.

### 3.6 CD312 Could Bind to GNA15

**FIGURE 6:**
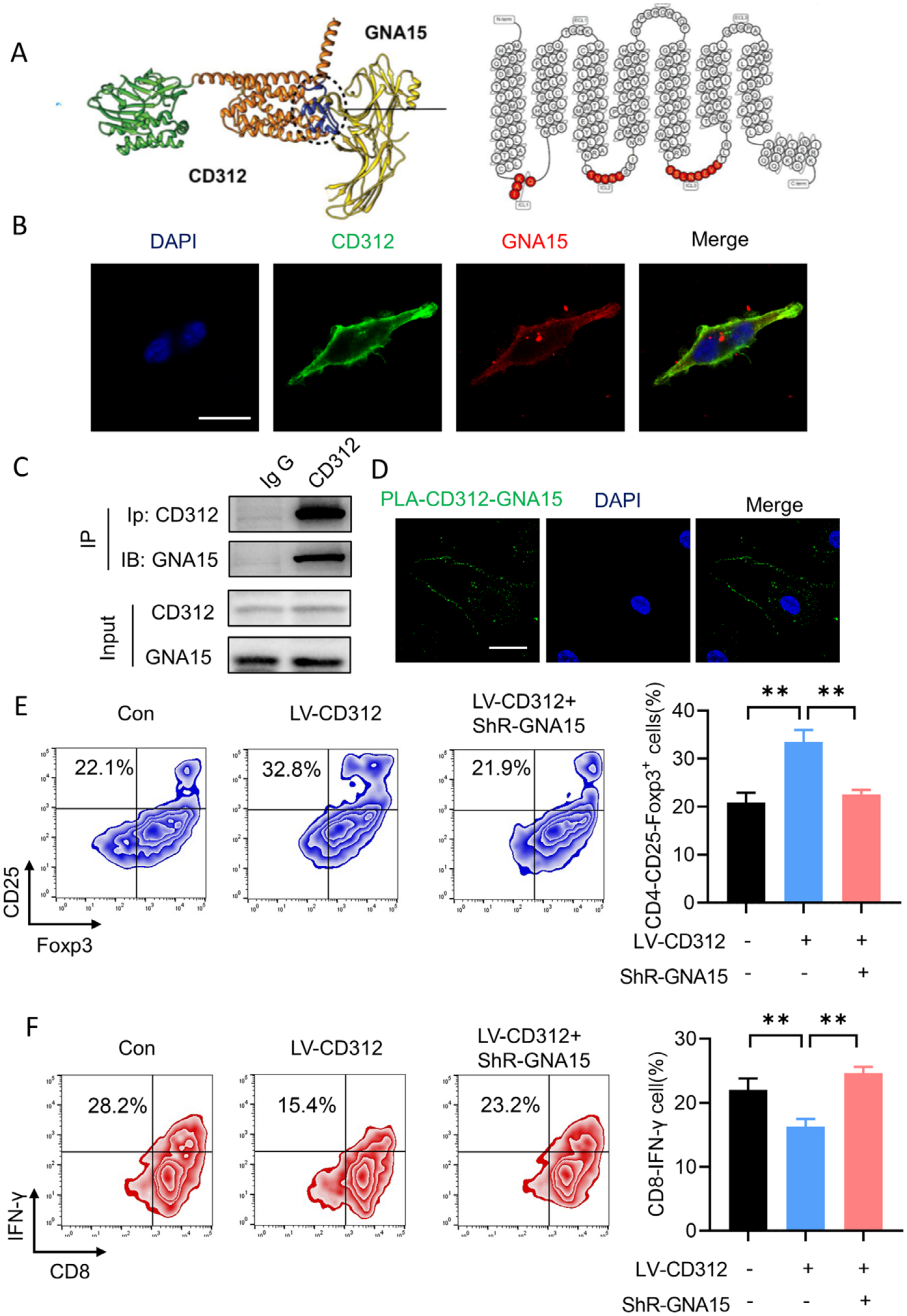
CD312 interacts with GNA15. (A) Prediction of potential binding sites between CD312 and GNA15. (B, C) Immunofluorescence assay and coimmunoprecipitation assay were performed to indicate the interaction between CD312 and GNA15. Scale bar = 20 μm. (D) PLA assay was used to detect the interaction between CD312 and GNA15. Scale bar = 20 μm. (E, F) The proportion of the CTL subgroup and Treg subgroup in immune cells was detected by flow cytometry upon CD312 overexpression and GNA15 knockdown or not. *n* = 3. **p < 0.01.

Finally, we explored the exact mechanism of CD312 in CD3+ T cells. Potential binding sites between CD312 and GNA15 were predicted. As shown in Figure 6A, CD312 may have good affinity with GNA15 in the transmembrane intracellular segment, and its specific binding sites are located in the intracellular loop region (red area). Immunofluorescence assays were performed to indicate the interaction between CD312 and GNA15 (Figure 6B). Co‐immunoprecipitation assays were also used to verify this interaction. Western blot assays confirmed that CD312 could interact with GNA15 (Figure 6C). The PLA assay showed similar results (Figure 6D). We then detected the distribution of Treg and CTL cell subpopulations through the CD312/GNA15 axis. GNA15 was knocked down in CD312‐overexpressed CD3+ T cells. The results indicated that the proportion of the Treg subgroup in immune cells was significantly upregulated upon CD312 overexpression, while the proportion of the Treg subgroup was decreased by GNA15 knockdown. Meanwhile, the proportion of the CTL subgroup in immune cells showed the opposite results (Figure 6E,F).

### 3.7 CD312 Promoted the Proliferation of Leukaemia Cells Through Binding to GNA15

**FIGURE 7:**
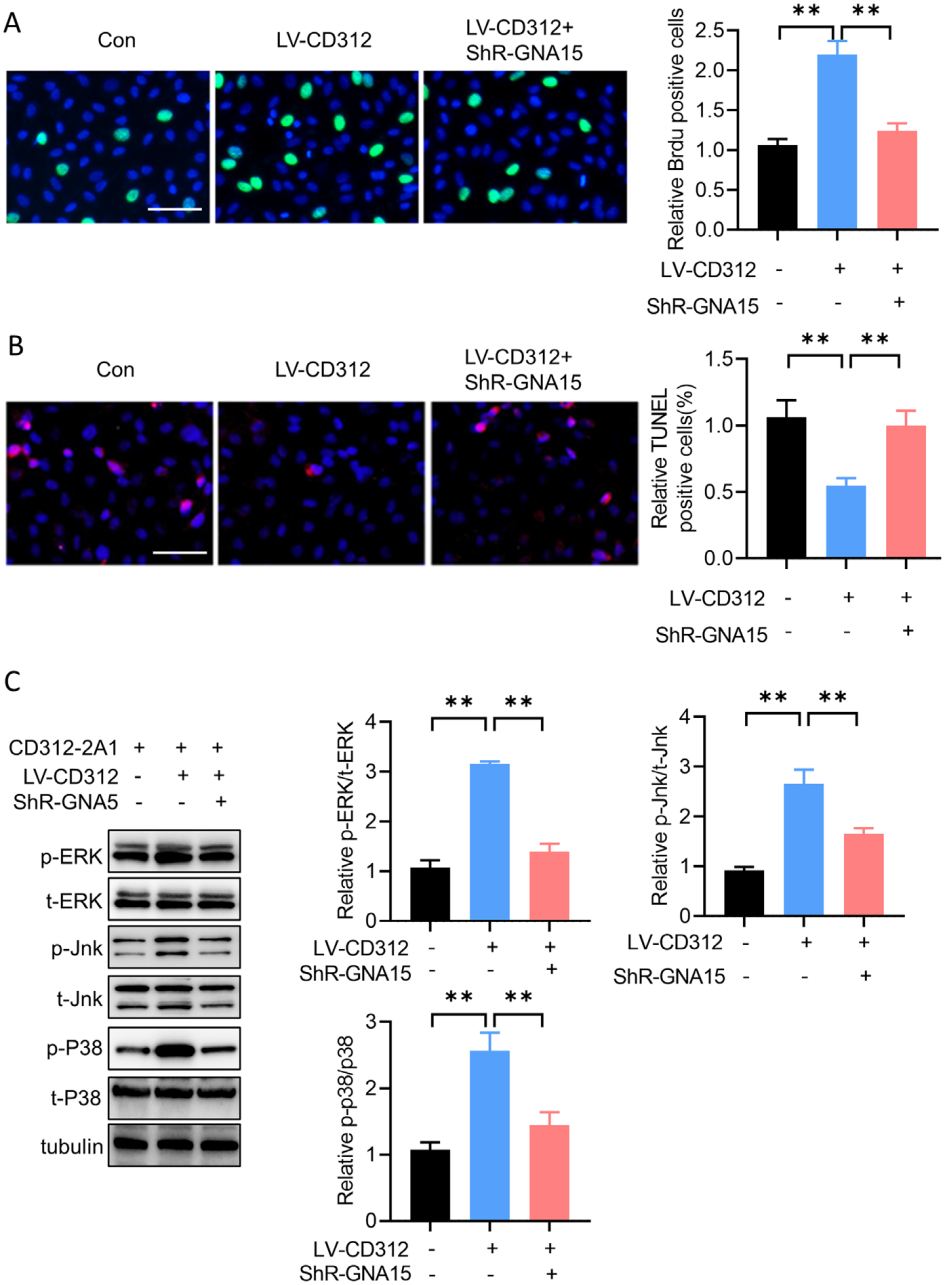
CD312 promoted the proliferation in leukaemia cells through binding to GNA15. (A) The BrdU staining assay was used to detect the proliferation of leukaemia cells, which was increased in the CD312 overexpressed CD3 + T cells group, while it was downregulated by GNA15 knockdown. *n* = 3. (B) The TUNEL staining assay was used to detect the apoptosis of leukaemia cells, which was increased in the CD312 overexpressed CD3 + T cells group, while it was downregulated by GNA15 knockdown. *n* = 3. (C) The phosphorylation of ERK, JNK and p38 was detected by western blot in CD312 overexpressed CD3 + T cells and GNA15 knockdown or not. *n* = 3. **p < 0.01.

To clarify whether GNA15 is involved in a CD312‐mediated bone marrow immune microenvironment, we used a co‐culture system to detect the proliferation and apoptosis of leukaemia cells. The BrdU staining assay showed that the proliferation of leukaemia cells was increased in the CD312‐overexpressed CD3+ T cells group, while it was downregulated by GNA15 knockdown (Figure 7A). Cell apoptosis was evaluated by TUNEL staining. The results indicated that cell apoptosis was decreased in the CD312‐overexpressed CD3+ T cells group, and this effect was cancelled by GNA15 knockdown (Figure 7B). The CD312 1A2 monoclonal antibody serves as a signal activator to convert CD312 into an activated state, but its effect on T cells is still unknown. We detected the signalling pathway in CD312 1A2‐treated cells. The results showed that the phosphorylation of ERK, JNK and p38 was increased in CD312‐overexpressed CD3+ T cells, but this effect was abolished by GNA15 knockdown (Figure 7C).

## 4 Discussion

For a long time, many studies on the mechanism of childhood ALL had focused on the characteristics of tumour cells themselves. However, the therapy based on tumour solid cells could not slow down the process of childhood ALL. The immune microenvironment composed of macrophages, lymphocytes and others is important in the occurrence and development of tumours [17, 18]. On the one hand, many immune cells directly participate in the inflammatory process and play a role in promoting inflammation and carcinogenesis [19]. On the other hand, the immune system also plays an immune monitoring function, clearing malignant transformed cells [20]. The immune microenvironment and molecular regulatory mechanisms in childhood ALL are very important.

GPCR also plays a crucial role in human inflammatory diseases [20, 21]. For example, CXCR1 and CXCR2 mainly expressed on neutrophils [22]. CXCL1, CXCL2 and CXCL8 produce a local inflammatory response when infection or injury occurs. At present, there are relatively few reports on the specific role of GPCR in the tumour immune microenvironment. We used the high‐throughput transcriptome sequencing to identify the differentially expressed genes in three bone marrow samples from ALL children and three bone marrow samples from children without non‐tumour diseases. It indicated that six GPCRs exhibited differential expression in both CD8^+^ and CD4^+^ T cells. The level of CD312 expression was decreased in CD8^+^ T cells, which was increased in CD4^+^ T cells. We also detected five other GPCR expressions. There was no significant statistical difference in FZD4, GPR135 and HRH1 between children with ALL and control groups. The LAPR1 and MTNR18 level showed a higher expression in CD4 + T cells. CD312 is also related to the distribution of Treg and CTL in the bone marrow immune microenvironment of ALL children. Additionally, the proportion of the Treg subgroup in immune cells was significantly downregulated, while the proportion of CTL subgroup cells was increased after CD312 knockdown.

CD312 is also known as ADGRE2 or EMR2. It has been found that CD312 protein is distributed on the surface of a variety of immune cells, such as Mast cell [23]. In addition, activated CD312 induces the expression of pro‐inflammatory mediators, including the activation of TNF‐ α and MMP‐9 [24]. The co‐culture system was constructed to detect the effects of CD312 in CD3^+^ T cells on leukaemia cells. The results showed that the expression of CD312 in CD3^+^ T cells could promote the proliferation of leukaemia cells. Additionally, the CD312‐mediating signalling pathway could be amplificated by Gα 16, resulting in activating AKT kinase, JNK pathway and NF‐ κB pathway in B cells [25]. CD312 contains 823 amino acids. In N‐terminal signal peptide, five Epidermal Growth Factor [EGF]‐like domains almost were identical to CD97 with multiple N and O glycosylation sites [26, 27]. The protein structure model was used to explore its potential binding sites between CD312 and Gα 16. The immunofluorescence assay and coimmunoprecipitation assay indicated the interaction between CD312 and GNA15. The PLA assay showed the same results. Then, it indicated that the proportion of the Treg subgroup in immune cells was significantly upregulated upon CD312 overexpression, while the proportion of the Treg subgroup was decreased by GNA15 knockdown. Meanwhile, the proportion of the CTL subgroup in immune cells showed the opposite results. However, we did not clarify the exact sequences between two proteins. The potential binding sites will be investigated for drug development. Then, we also indicated that the phosphorylation of ERK, JNK and p38 was increased in CD312‐overexpressed CD3^+^ T cells; however, the effect was abolished by GNA15 knockdown. It indicated that GNA15 is involved in a CD312‐mediated bone marrow immune microenvironment. The high‐throughput transcriptome sequencing also needs to identify the novel signalling pathway.

Indeed, there are some limitations in our study. Firstly, we did not conduct in vivo experiments; an ideal model would involve conditional knockout mice for CD312 to validate its impact on immune cell function. Additionally, future research needs to further explore other signalling pathways that may interact with CD312, clarifying its specific regulation of downstream targets. This will provide a stronger theoretical foundation for the development of targeted therapies against CD312.

In conclusion, we showed that the level of CD312 expression was decreased in CD8^+^T cells, which was increased in CD4^+^ T cells. The expression of CD312 is related to the distribution of Treg and CTL in the bone marrow immune microenvironment of ALL children. Moreover, CD312 could bind to GNA15 in CD3^+^ T cells to promote the proliferation in leukaemia cells through activating the phosphorylation of ERK, JNK and p38 pathway.

## Author Contributions

**Yaping Wang:** conceptualization (equal), funding acquisition (equal), investigation (equal), methodology (equal), project administration (equal), validation (equal), writing – review and editing (equal). **Jiali Wang:** data curation (equal), investigation (equal), methodology (equal), writing – original draft (equal). **Xiaopeng Ma:** data curation (equal), methodology (equal), validation (equal). **Huimin Li:** formal analysis (equal), visualization (equal). **Xiaoyan Sun:** methodology (equal), writing – original draft (equal). **Meiyun Kang:** investigation (equal). **Heng Zhang:** validation (equal), writing – review and editing (equal). **Yao Xue:** data curation (equal), formal analysis (equal). **Yongjun Fang:** data curation (equal), funding acquisition (equal), project administration (equal), writing – review and editing (equal).

## Conflicts of Interest

The authors declare no conflicts of interest.

## Supporting information


**Figure S1.** Five GPCR expressions in both CD8^+^ T cells and CD4^+^ T cells. (A–C) The expression of FZD4, GRP135 and HRH1 is not significant between two groups. (D, E) The LAPR1 and MTNR18 level showed a higher expression in CD4 + T cells. *n* = 10. **p < 0.01, ***p < 0.001.

## Data Availability

The original data presented in the study are included in the article, which are available from the corresponding author upon reasonable request. RNA sequencing data reported in this paper are available in the ArrayExpress database (accession numbers E‐MTAB‐13176 and E‐MTAB‐13177).
