# Correction to “CD312 Promotes Pediatric Acute Lymphoblastic Leukemia Through GNA15 Mediated Non‐Classical GPCR Signaling Pathway”

**DOI:** 10.1111/jcmm.70684

**Published:** 2025-07-24

Wang Y, Wang J, Ma X, Li H, Sun X, Kang M, Zhang H, Xue Y, Fang Y., “CD312 Promotes Paediatric Acute Lymphoblastic Leukaemia Through GNA15‐Mediated Non‐Classical GPCR Signalling Pathway.” Journal of Cellular and Molecular Medicine. 2024 Dec;28(23):e70283. doi: 10.1111/jcmm.70283.

In Wang Y et al., there were errors in Figure 1, 3, 4 and 6. The distribution of Treg and CTL populations in human bone marrow samples in Figure 1B; the effect of CD312 on the distribution of Treg and CTL in Figures 3A and 3B; the impact of CD312 knockdown on Treg and CTL distribution in Figures 4C and 4D; and the rescue experiments involving CD312 and GNA15 in Figures 6E and 6F were affected. The corrected figures are presented below. The authors confirm that all results and conclusions of this article remain unaffected.

**FIGURE 1:**
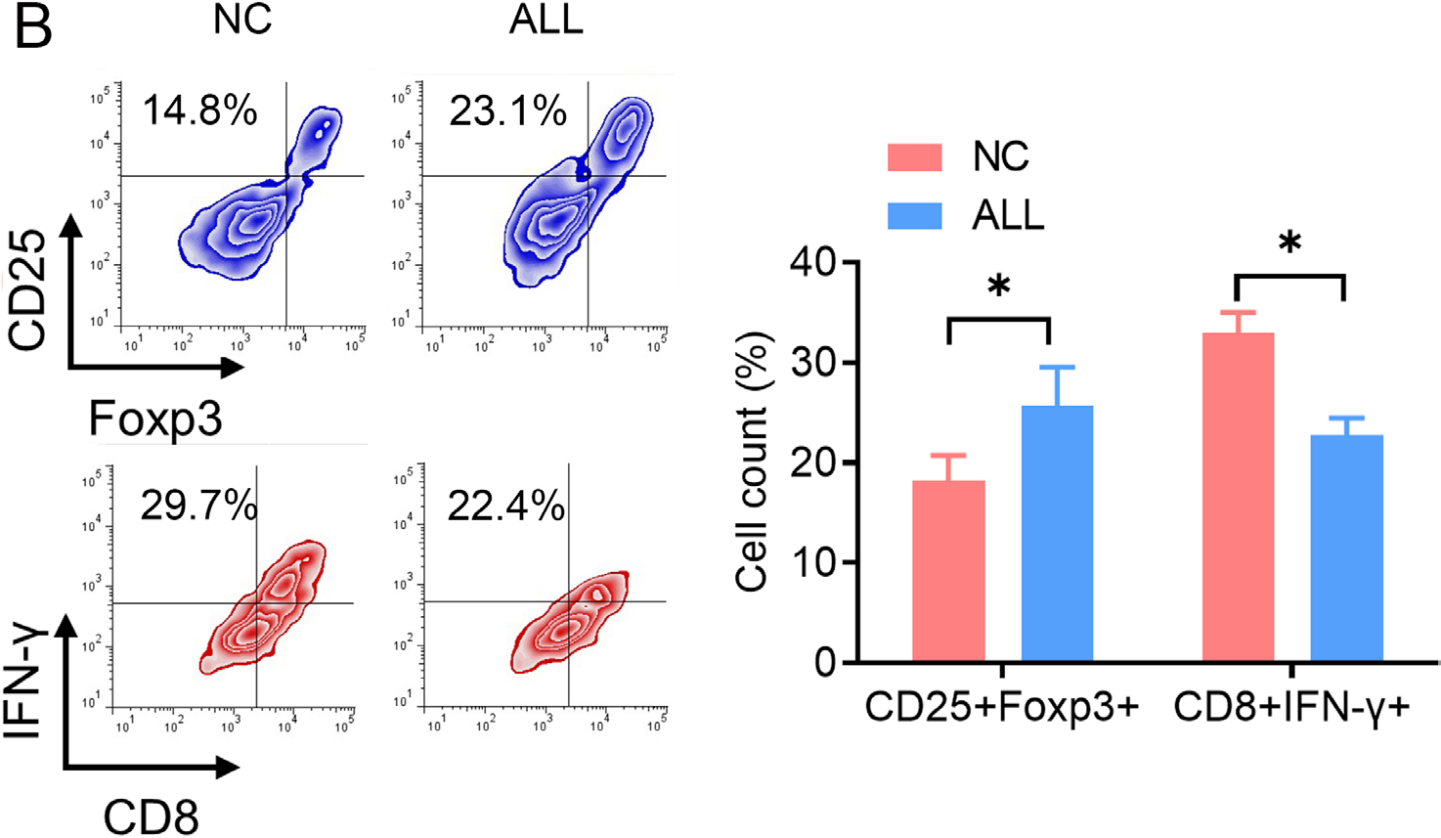
The expression of GPCRs in ALL children. (B) Flow cytometry was used to observe the distribution of Treg cell subpopulations and CTL cell subpopulations in the bone marrow. *n* = 5.

**FIGURE 3:**
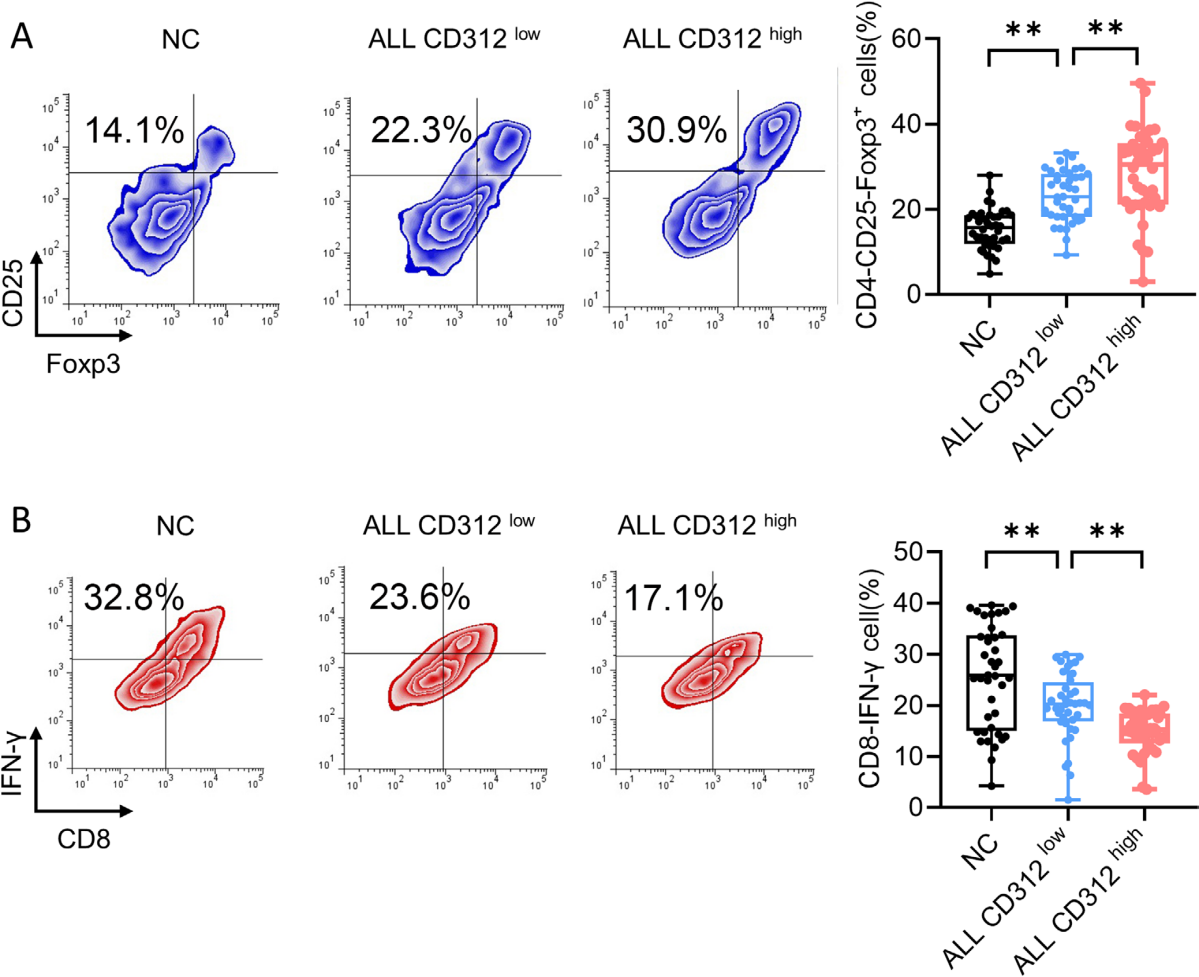
CD312 was related to the distribution of Treg and CTL cells. (A) The proportion of Treg cells was detected by flow cytometry in the normal control group, CD312 high expression group (CD312 high) and CD312 low expression group (CD312 low). *n* = 40. (B) The proportion of CTL cells was detected by flow cytometry in the normal control group, CD312 high expression group (CD312 high) and CD312 low expression group (CD312 low). *n* = 40. **p < 0.01.

**FIGURE 4:**
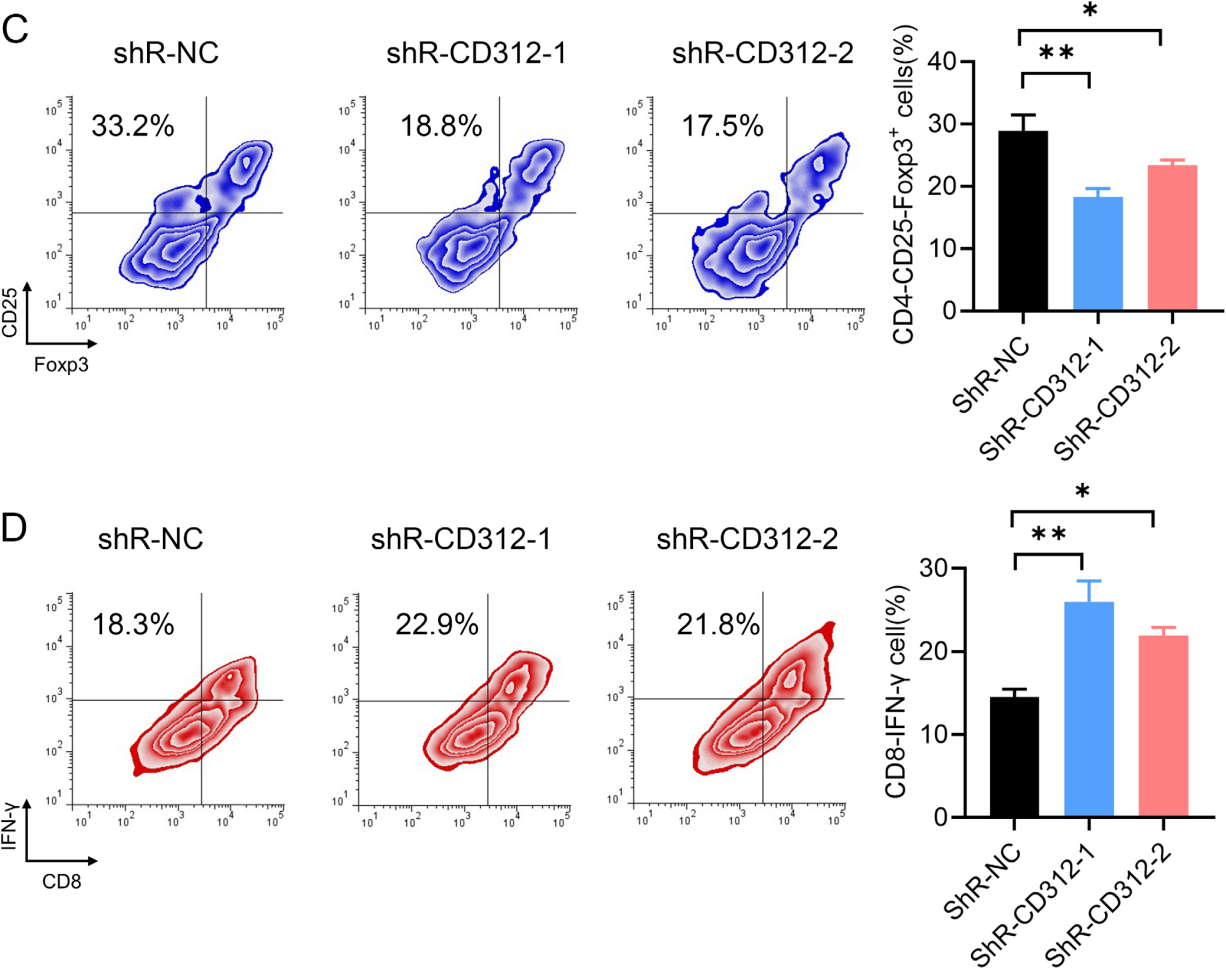
CD312 knockdown regulated Treg and CTL cells distribution. (C, D) The distribution of Treg and CTL in the cell subpopulations was detected by flow cytometry after CD312 knockdown.

**FIGURE 6:**
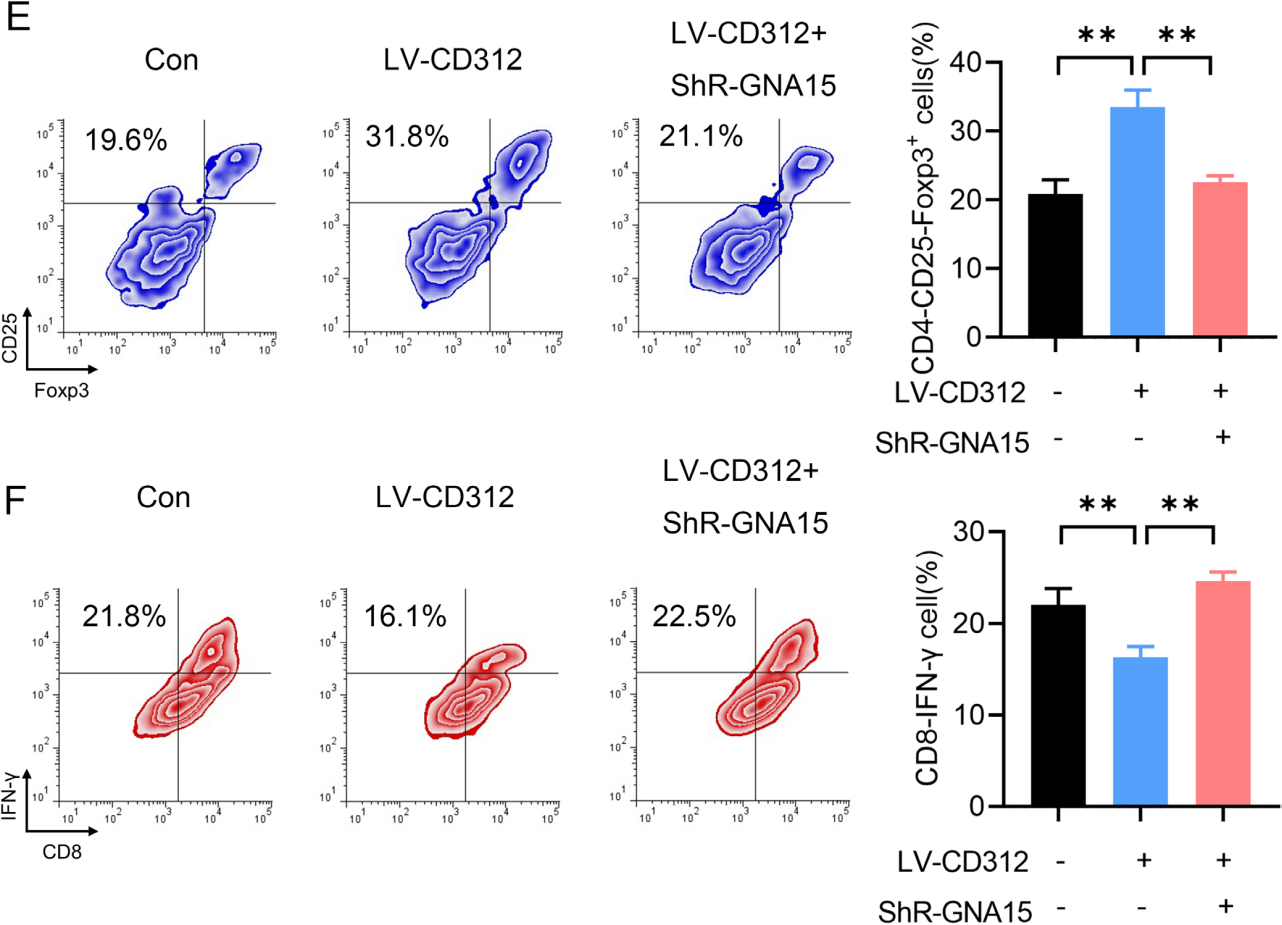
CD312 interacts with GNA15. (E, F) The proportion of the CTL subgroup and Treg subgroup in immune cells was detected by flow cytometry upon CD312 overexpression and GNA15 knockdown or not.

