# Supplementary material for: CD312 Promotes Paediatric Acute Lymphoblastic Leukaemia Through GNA15‐Mediated Non‐Classical GPCR Signalling Pathway
Source: J Cell Mol Med. 2024 Dec 10;28(23):e70283. doi: 10.1111/jcmm.70283 (PMC11629795; doi:10.1111/jcmm.70283)
Supplement: Supplementary file 1 — Figure S1. Five GPCR expressions in both CD8+ T cells and CD4+ T cells. (A–C) The expression of FZD4, GRP135 and HRH1 is not significant between two groups. (D, E) The LAPR1 and MTNR18 level showed a higher expression in CD4 + T cells. n = 10. **p < 0.01, ***p < 0.001. [file JCMM-28-e70283-s001.docx]

***Supplementary material***

**CD312 promotes pediatric acute lymphoblastic leukemia through GNA15 mediated non-classical GPCR signaling pathway**

Yaping Wang^1,#,^ *, Jiali Wang^1,#^, Xiaopeng Ma^1^, Huimin Li^1^, Xiaoyan Sun^1^, Meiyun Kang^1^,Heng Zhang^1^, Yao Xue^1^, Yongjun Fang^1,^ *

1. Department of Hematology and Oncology, Children's Hospital of Nanjing Medical University, Nanjing Medical University, 72# Guangzhou Road, Nanjing, Jiangsu Province, China.

^#^ These authors contributed equally to this work.

*Corresponding authors:

Dr. Yongjun Fang, Department of Hematology and Oncology, Children's Hospital of Nanjing Medical University, Nanjing Medical University, 72 Guangzhou Road, Nanjing, Jiangsu Province, China.

Phone: 86-25-51554586, Fax: +86-25-83304239

E-mail: [fyj322@189.cn](mailto:dryjfang@gmail.com)

Dr. Yaping Wang, Department of Hematology and Oncology, Children's Hospital of Nanjing Medical University, Nanjing Medical University, 72 Guangzhou Road, Nanjing, Jiangsu Province, China.

Phone: 86-25-51554586, Fax: +86-25-83304239

E-mail: [wyp_0919@163.com](mailto:wyp_0919@163.com)

**
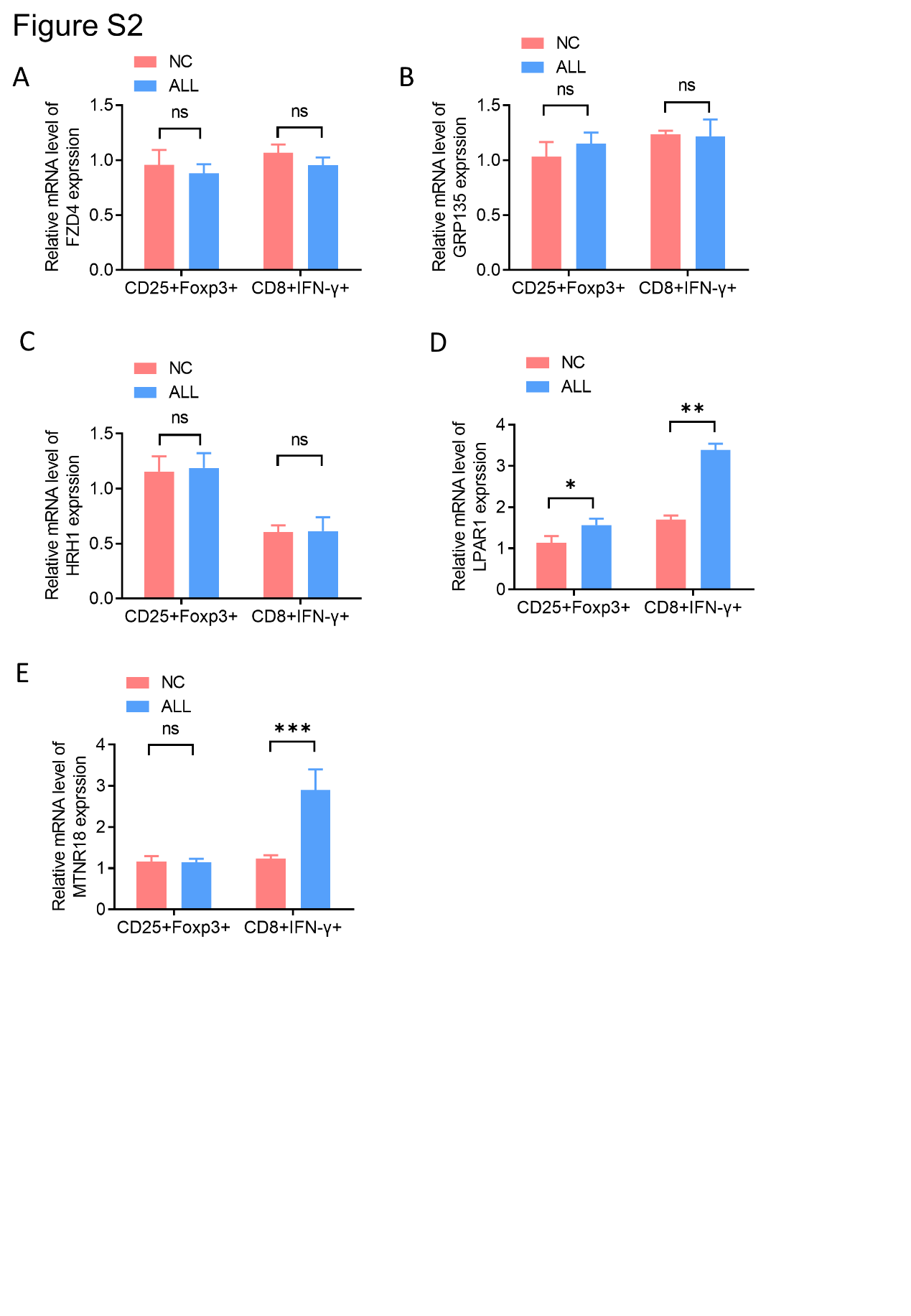
FigureS1**

**Figure S1. 5 other GPCR expression in in both CD8^+^ T cells and CD4^+^ T cells.** A-C. The expression of FZD4, GRP135 and HRH1 is not significant between two groups. D-E. The LAPR1 and MTNR18 level was higher expression in CD4+T cells. n=10. **p<0.01. ***p<0.001.
